# Sidedness and Molecular Pattern in Defining the Risk of Lymph Node Metastasis in Nonmetastatic Colorectal Cancer: Single-Center Retrospective Study

**DOI:** 10.3390/cancers16193314

**Published:** 2024-09-27

**Authors:** Edoardo Maria Muttillo, Francesco Saverio Li Causi, Alice La Franca, Alessio Lucarini, Giulia Arrivi, Leonardo Di Cicco, Giorgio Castagnola, Andrea Scarinci, Federica Mazzuca, Genoveffa Balducci, Paolo Mercantini

**Affiliations:** 1 Department of Medical Surgical Science and Translational Medicine, Sant’Andrea Hospital, Sapienza University of Rome, 00198 Rome, Italy; edoardomaria.muttillo@uniroma1.it (E.M.M.); alice.lafranca@uniroma1.it (A.L.F.); alessio.lucarini@uniroma1.it (A.L.); leonardodicicco1@gmail.com (L.D.C.); giorgiocastagnola@hotmail.com (G.C.); andreascarinci82@hotmail.com (A.S.); genoveffa.balducci@uniroma1.it (G.B.); paolo.mercantini@uniroma1.it (P.M.); 2 Department of Digestive Surgery, Hopital Edouard Herriot, 69003 Lyon, France; 3 Oncology Unit, Department of Clinical and Molecular Medicine, Sant’Andrea University Hospital, Sapienza University of Rome, 00189 Rome, Italy; giulia.arrivi@uniroma1.it (G.A.); federica.mazzuca@uniroma1.it (F.M.)

**Keywords:** colorectal cancer, cancer surgery, cancer oncology, cancer biology, cancer histology, lymphadenectomy, CME

## Abstract

**Simple Summary:**

Histopathological and molecular factors could play a role in determining LNM in colon cancer. Extended lymphadenectomies in right-sided patients have been associated with complications. We demonstrated that histopathological and molecular analysis can be useful in predicting LNM and therefore identify high risk patients which could potentially benefit from D3 lymphadenectomy/CME.

**Abstract:**

**Background**: Lymphadenectomy plays a central role in the treatment of localized colon cancer. While in left colon cancer the D3 lymphadenectomy/CME is considered the standard of care, lymphatic stations to be removed in right colon cancer are still a matter of discussion. The individuation of LNM risk factors could help in choosing the lymphadenectomy in right-sided tumors. This study aims to analyze the correlation of histopathological and molecular characteristics with lymph node metastasis, both in right- and left-sided colon cancer, and their impact on survival; **Methods**: We conducted a single-center observational retrospective study. The following data were collected and analyzed for each patient: demographics, histopathological and molecular data, and intraoperative and perioperative data. Statistical analyses were performed, including descriptive statistics, multivariate logistic regression and survival analysis; **Results**: An association between tumor size (pT, *p* < 0.001), grading (*p* = 0.013), budding (*p* < 0.001), LVI (79,4% *p* < 0.001) and LNM was observed. A multivariate analysis identified pT4 (OR 5.45, *p* < 0.001) and LVI+ (OR 10.7, *p* < 0.001) as significant predictors of LNM. Right-sided patients presented a worse OS when associated with LNM, while no significant difference was observed in N0 patients; **Conclusions**: histological and molecular analysis can help identify high risk patients, which could benefit from extended lymphadenectomies. These patients could be ideal candidates for the D3 lymphadenectomy/CME.

## 1. Introduction

Colorectal cancer (CRC) is the third most commonly diagnosed malignancy, and the second most common cause of cancer-related death [1].

Despite often being considered as a single identity, right- and left-sided colon cancers constitute two distinguished entities in terms of anatomy, vascularization, mutational profile and, when in an advanced stage, also in terms of prognosis [2,3].

Neoplasms of the right colon are, in other words, biologically different from those of the left colon [4,5], and those differences can be taken into consideration when we approach their treatment.

Surgery remains a cornerstone in the treatment of both right- and left-sided localized colon cancer [4,5,6], and lymphadenectomy plays a central role: a correctly executed lymphadenectomy is, in fact, not only important in allowing a correct stadiation of the disease, but it is important also in terms of prognosis and future treatment planning [6,7,8]. However, while in left-sided colon cancer there is a strong consensus on the D3 lymphadenectomy/CME being the standard of care for resectable tumors, lymphatic stations to be removed in patients with right-sided colon cancer are still a matter of discussion [6,7,8], with Asian guidelines advocating extended lymph node removal (D3) on a routine basis in T3/T4 and selected T2 stages [9], while Western guidelines recommend the execution of the D2 lymphadenectomy only [6,7,8].

These differences are strongly connected to the vascular anatomy of these tumors: in the left colon, the possibility of ligating the inferior mesenteric pedicles at the origin makes the D3 lymphadenectomy/CME simple and safe to execute. On the contrary, in the right colon, the impossibility of ligating the superior mesenteric pedicles makes the D3 lymphadenectomy/CME more challenging. The D3 lymphadenectomy in right-sided colon cancer has been associated with a higher risk of vascular complications [10], despite it being associated with some survival advantages [11], and is therefore not only generally not recommended by Western guidelines, but also not routinely executed in high-volume centers in Italy [12].

Histological and molecular factors can have an impact on lymph node metastatization. This relation was first studied in colorectal cancer by Ueno et al. [13], who conducted a study on 292 patients with invasive colon cancer, and more recently confirmed by Yasue et al. [14], who conducted a similar study on 846 cases of T1 stage colorectal cancer.

This study aims to analyze the correlation of histopathological and molecular characteristics with lymph node metastasis, both in right- and left-sided colon cancer (primary endpoint). This study also aims to analyze the survival of patients with colon cancer and with or without lymph node metastasis (secondary endpoint).

## 2. Materials and Methods

A retrospective single-center study has been conducted on patients diagnosed with resectable colon cancer at our institution who underwent surgical resection between January 2019 and December 2023. These patients were identified from a prospectively maintained institutional database.

### 2.1. Patients Selection

**Figure 1:**
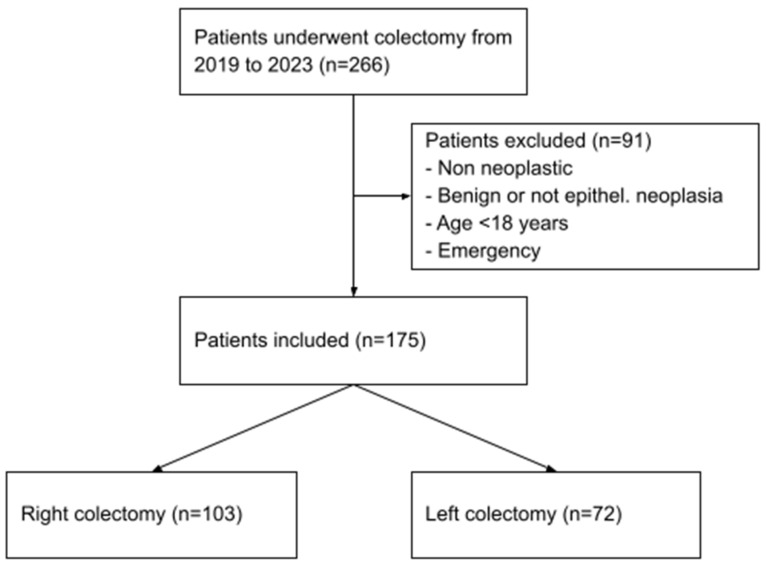
Patients selection process.

Patients with benign or non-neoplastic histology were excluded, as well as patients aged less than 18 years old. Other exclusion criteria were pregnancy at the time of the resection and emergency surgeries. Out of the 266 patients initially identified, 91 were excluded because they did not meet the inclusion criteria, resulting in a final population of 175 cases (Figure 1).

### 2.2. Analyzed Features and Surgical Technique

Disease was staged according to the indications of the American Joint Committee on Cancer (AJCC) Cancer Staging Manual, 8th edition. Histopathological and molecular data were extracted from histological reports, and the features analyzed were as follows: histotype, tumor T stage (pT), tumor N stage (pN), histological grade of differentiation (G), tumor budding (Bd), lymphovascular invasion (LVI) and microsatellite status (microsatellite instability (MSI) or microsatellite stability (MSS)). Colorectal cancers demonstrating the MSI phenomenon were further divided into two distinct MSI tumor phenotypes: MSI-high (MSH-H) and MSI-low (MSI-L), while tumors that did not present evidence of budding were indicated as Bd0.

Surgical resection was conducted according to the standards adopted by our institution by experienced surgeons. In right colon cancers, a D2 lymphadenectomy was performed, while in left colon cancer, a D3 lymphadenectomy associated with CME was performed.

### 2.3. Statistical Analysis

Continuous variables are presented as the mean and standard deviation (SD), while categorical variables are expressed as units and percentages. Descriptive statistics were used to summarize information relevant to the study. The differences between groups were analyzed using Student’s *t*-test for continuous variables, and Pearson’s Chi-squared test or Fisher’s exact test, as appropriate, for categorical variables. A multivariate binomial logistic regression was created to identify independent predictors of outcomes. A survival analysis was conducted according to the Kaplan–Meier method. Differences in survival curves were evaluated by adopting the log-rank test. Significance was accepted at *p* < 0.05.

All statistical analyses were conducted using R (version 4.3.1; R Foundation for Statistical Computing, Vienna, Austria).

## 3. Results

### 3.1. Clinical Characteristics of Patients

Of the 175 patients identified, 105 were males (60%) and 70 females (40%). The mean age was 74 (SD = 11). Most of the patients presented an American Society of Anesthesiologists (ASA) score of II (*n* = 43, 25%) or greater. Most of the patients in our cohort had a TNM stage of III (*n* = 36, 21%) or IV (*n* = 14, 8%). The most frequent localizations were ascending colon (*n* = 75, 43%) and sigmoid colon (*n* = 33, 19%). Right-sided colon cancer patients were significantly older (*p* = 0.048). Baseline characteristics are reported in Table 1.

### 3.2. Operative Results

The mean operative time was 202 min (SD = 55). The majority of the procedures were conducted video-laparoscopically (*n* = 121, SD = 55). Left colectomies presented a significantly longer operative time (212.2 vs. 194.9, *p* = 0.040) and were associated with a higher rate of stoma formation (*p* = 0.002). The operative results are summarized in Table 2.

### 3.3. Histopathological and Molecular Features

The mean lymph node yield was 19.3 (SD = 8.5), with a significantly higher number of retrieved lymph nodes in right hemicolectomy specimens (20.8 vs. 17.3, *p* = 0.007). The R0 rate was 100%. Histological variables (pT, pN, G Bd, LVI) were all comparable except for tumor grading (G, *p* = 0.028). Left-sided tumors showed a higher incidence of MSS (*p* < 0.001). Most of the patients had an MSS molecular profile (*n* = 89, 50.9%), as shown in Table 3.

**Figure 2:**
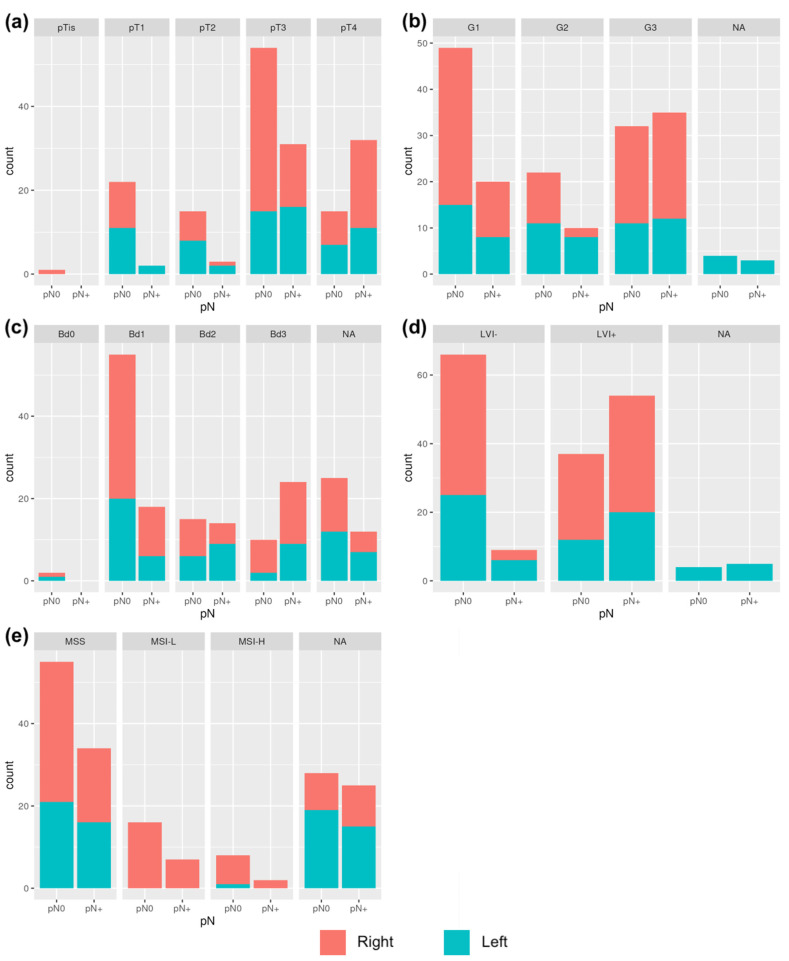
Variables distributions in relation to LNM in right- and left cancers: (**a**) correlation between LNM and pT; (**b**) correlation between LNM and G; (**c**) correlation between LNM and Bd; (**d**) correlation between LNM and LVI; (**e**) correlation between LNM and MSI.

When comparing the pN0 and pN+ patients, all of the histological features showed statistically significant differences in variable distributions. These differences were also found when analyzing right- and left-sided patients separately, as presented in Table 4 and Figure 2.

A multivariate analysis showed a significant correlation between lymph node metastasis and lymphovascular invasion (LVI), both in the general population (OR = 20.64, *p* < 0.001) and in the right-sided and in the left-sided population separately (*p* = 0.006 and *p* = 0.041 for right- and left-sided patients, respectively). In right-sided patients, T4 was also found to be a significant predictor of LNM (OR = 8.14, *p* = 0.019), with near significance in the general population (Table 5).

### 3.4. Survival Analysis

**Figure 3:**
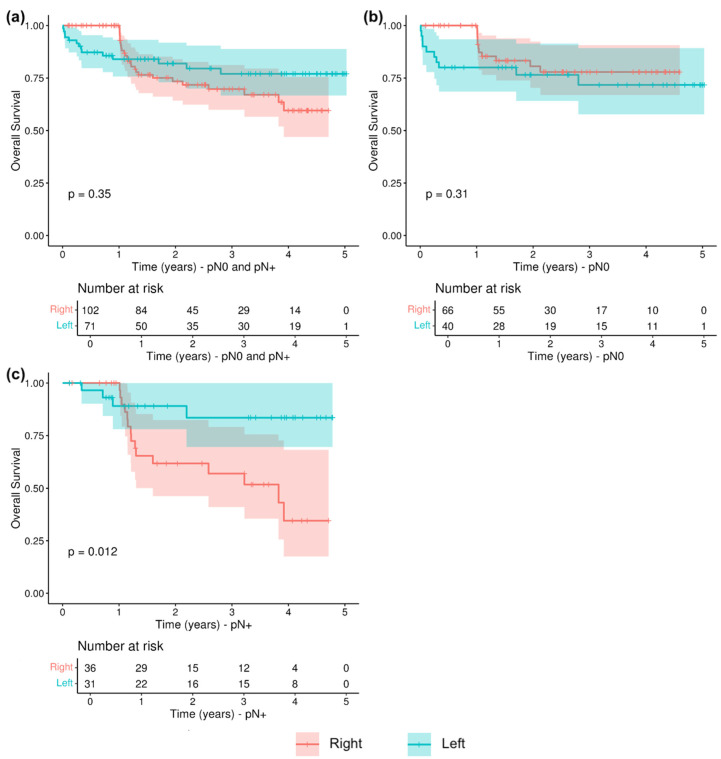
Overall survival (OS) in relation to LNM and tumor sidedness: (**a**) OS in pN0 and pN+ patients; (**b**) OS in pN0 patients; (**c**) OS in pN+ patients.

A Kaplan–Meier analysis showed no significant differences in terms of overall survival (OS) between right- and left-sided patients (*p* = 0.35, Figure 3a). However, when the population was grouped according to lymph nodes positivity, right-sided colon cancers with LNM showed a significantly worse prognosis than left-sided colon cancers with LNM (*p* = 0.012, Figure 3b), while there were no significant differences between right- and left-sided patients without nodes involvement (*p* = 0.31, Figure 3c).

## 4. Discussion

This study showed that lymph node metastases determine a greater prognosis worsening in right-sided colon cancer (RSCC) compared to left-sided colon cancer (LSCC), suggesting the need for a change of perspective in the surgical approach. This investigation of predictive factors has identified specific histopathological features: pT stage, grading, budding and lymphovascular invasion (LVI+).

Despite the absence of any difference in terms of overall survival (OS) between RSCC and LSCC, survival analysis stratified by lymph node staging revealed a significantly worse prognosis of RSCC in the presence of lymph node metastasis (*p* = 0.012). This interesting finding could be explained by the different approaches of surgical lymphadenectomy. In fact, the standard technique of right hemicolectomy does not involve lymphadenectomy of the mesenteric–celiac axis; on the other hand, left hemicolectomy always includes in these cases the high ligation of the inferior mesenteric artery and thus ensures, if properly performed, local lymph nodal radicality.

A univariate analysis conducted to investigate risk factors for lymph node involvement showed that pT stage, grading, budding and LVI+ had significant correlation with lymph node metastasis, but a multivariate analysis identified pT4 stage and LVI+ as predominant independent predictors, according to ESMO guidelines for stage II that recognize node retrieval and T4 as major risk factors [6]. Moreover, this association appeared to be much stronger in RSCC (OR 8.19 and 25.96, respectively, for pT4 and LVI+).

To our knowledge, the literature on this topic appears limited. The role of histopathologic-molecular factors was studied by Ueno et al. [13] on 292 patients with invasive colon cancer to identify the role of grading, budding and LVI in lymph nodal metastasis. More recently, Yasue et al. [14] conducted a similar analysis on 846 cases of T1-stage colorectal cancer. In this study, however, it was decided to analyze RSCC and LSCC as two separate entities, namely intermediate- and advanced-stage neoplasms.

An analysis of microsatellite stability yielded interesting results. MSI appeared to be a protective factor against lymph nodal invasion (OR < 1), but it did not seem to influence the prognosis in the pN0 patients. However, in the pN+ group, the survival curve of MSS/MSI-Low patients showed a steep drop, suggesting that MSS provides a considerable disadvantage in terms of prognosis in the presence of lymph node metastasis.

Hakki et al. [15] analyzed 1466 cases of colon cancer, of which 361 (25%) were MSI. In these patients, pN+ status was associated with pT stage, LVI+ and MSS.

In view of the above, while in LSCC the current standard surgical technique appears adequate regardless of histologic-molecular arrangement and lymph nodal status, in RSCC a change in perspective to identify two different groups of patients appears needed. Considering the proven importance of proper lymphadenectomy for oncological purposes [16,17], in patients considered “low risk” (MSI and without preoperatory signs of lymph nodal involvement, cN0), the current standard right hemicolectomy technique seems adequate; meanwhile, “high-risk” patients (MSS with cN+) could be appropriate candidates for right hemicolectomy with complete mesocolic excision (CME). Indeed, this procedure is still controversial due to the technical difficulty and the increased complication rate encountered.

In 2022, three criteria for defining CME were established by the Portsmouth Consensus Statements [18]: (1) central vascular ligation, (2) exposure of the superior mesenteric vein (SMV) and (3) excise ion of an intact mesocolon.

Regarding technical feasibility and short-term outcomes, a controlled trial conducted in 2020 on 330 patients [19] showed a greater number of harvested lymph nodes (24 vs. 20, *p* = 0002) in the CME group, without significant differences in terms of postoperative complications. CME patients also showed a lower rate of recurrence (0% vs. 9.8%, *p* < 0.001). These positive results were confirmed by a systematic review published in 2021 [20], which included 16 studies. However, the complexity of this technique was confirmed by the results of the CODIG-2 trial published in 2024 [12]: only 37.6% of surgical specimens were type 0 according to the Benz score [21].

In this light, the sensitivity and specificity of the radiological means at our disposal in preoperative staging appear crucial. Current CT techniques facilitate the detection of lymph node metastases with sensitivity and specificity of 71% (CI 59–81%) and 67% (CI 46–83%), respectively, according to the results of a recent meta-analysis [22]. The radiological diagnosis of lymph node invasion still has unresolved critical issues: the most accepted definition of lymph node positivity is the presence of a lymph node larger than 10 mm or a cluster of three or more lymph nodes regardless of size. This approach, however, exposes the risk of false positives (enlarged inflammatory lymph nodes) and false negatives (lymph nodes with micrometastases). The use of radiomics, in this scenario, could be promising to identify lymph node positivity in colorectal cancer. It was first proposed by Huang et al. [23], and in recent studies has been proven to be more precise than conventional techniques [24]; in 2022, Zhao et al. [25] also validated a deep learning model for predicting lymph node metastasis in colorectal cancer with encouraging results. However, stronger data on a larger population are still needed. From a molecular perspective, specific patterns currently studied mainly in metastatic disease (like mutations of KRAS and NRAS genes) [6,26,27] could be a useful tool for selecting high-risk patients. The importance of these factors was confirmed by Roth et al. [28], who analyzed their role in terms of overall survival from 1404 cases in the PETACC3 trial [29].

This study has several limitations: first, its observational and retrospective nature; second, the limited, although well-selected, sample (175 cases), which makes it necessary to confirm these results on a large scale, possibly with a multicenter study which is currently ongoing. We also excluded data regarding perineural invasion, and tumor-infiltrating lymphocytes due to the lack of homogenous reporting in our pathology exams. Finally, the survival analysis was conducted on a population that did not undergo a uniform follow-up period.

## 5. Conclusions

In conclusion, lymph nodal status in patients with right-sided colon cancer (RSCC) seems to have a stronger impact on survival compared with left-sided (LSCC), with RSCC N+ patients having a dramatic worsening of the prognosis (*p* = 0.012). In a multivariate analysis, pT4 stage and LVI+ have been shown to be independent predictors for lymph node metastasis in RSCC (OR = 25.96 and 8.19, respectively). Microsatellite instability (MSI) appeared to be a protective factor. Considering the impact of lymph nodal involvement on prognosis, likely due to the absence of a standard and radical lymphadenectomy in right hemicolectomy, this group of patients with pT4 stage, LVI+ and MSS status might be ideal candidates for CME.

## Figures and Tables

**Table 1:** Patients characteristics.

Variable		Right	Left	Total	*p*
Age	Mean (SD)	75.0 (11.0)	71.7 (10.2)	73.6 (10.8)	0.048
Sex	Female	40 (57.1)	30 (42.9)	70 (100)	0.826
	Male	63 (60.0)	42 (40.0)	105 (100)	
	(Missing)	0 (NaN)	0 (NaN)	0 (100)	
ASA	I	2 (100.0)	0 (0.0)	2 (100)	0.397
	II	24 (55.8)	19 (44.2)	43 (100)	
	III	66 (57.4)	49 (42.6)	115 (100)	
	IV	11 (73.3)	4 (26.7)	15 (100)	
	(Missing)	0 (NaN)	0 (NaN)	0 (100)	
TNM Stage	0	2 (100.0)	0 (0.0)	2 (100)	0.251
	I	18 (50.0)	18 (50.0)	36 (100)	
	II	44 (67.7)	21 (32.3)	65 (100)	
	III	31 (53.4)	27 (46.6)	58 (100)	
	IV	8 (57.1)	6 (42.9)	14 (100)	
	(Missing)	0 (NaN)	0 (NaN)	0 (100)	
Location	Caecum	12 (100.0)	0 (0.0)	12 (100)	<0.001
	Ascending	75 (100.0)	0 (0.0)	75 (100)	
	Hepatic flexure	4 (100.0)	0 (0.0)	4 (100)	
	Transverse	12 (100.0)	0 (0.0)	12 (100)	
	Splenic flexure	0 (0.0)	4 (100.0)	4 (100)	
	Descending	0 (0.0)	24 (100.0)	24 (100)	
	Sigmoid	0 (0.0)	33 (100.0)	33 (100)	
	Junction	0 (0.0)	11 (100.0)	11 (100)	
	(Missing)	0 (NaN)	0 (NaN)	0 (100)	

**Table 2:** Operative results.

Variable		Right	Left	Total	*p*
Operative time (min)	Mean (SD)	194.9 (51.1)	212.2 (58.7)	202.0 (54.9)	0.040
Technique	Open	29 (28.2)	20 (27.8)	49 (28.0)	0.685
	VLS	72 (69.9)	49 (68.1)	121 (69.1)	
	Robotic	2 (1.9)	3 (4.2)	5 (2.9)	
	(Missing)	0 (NaN)	0 (NaN)	0 (100)	
Associated procedure	No	94 (91.3)	67 (93.1)	161 (92.0)	0.883
	Yes	9 (8.7)	5 (6.9)	14 (8.0)	
	(Missing)	0 (NaN)	0 (NaN)	0 (100)	
Stoma formation	No	103 (100.0)	18 (25.0)	121 (69.1)	0.002
	Yes	0 (0.0)	3 (4.2)	3 (1.7)	
	(Missing)	0 (0.0)	51 (70.8)	51 (29.1)	

**Table 3:** Histopathological and molecular features.

Variable		Right	Left	Total	*p*
pT	pTis	1 (100.0)	0 (0.0)	1 (100)	0.303
	pT1	11 (45.8)	13 (54.2)	24 (100)	
	pT2	8 (44.4)	10 (55.6)	18 (100)	
	pT3	54 (63.5)	31 (36.5)	85 (100)	
	pT4	29 (61.7)	18 (38.3)	47 (100)	
	(Missing)	0 (NaN)	0 (NaN)	0 (100)	
pN	pN0	66 (61.7)	41 (38.3)	107 (100)	0.427
	pN+	37 (54.4)	31 (45.6)	68 (100)	
	(Missing)	0 (NaN)	0 (NaN)	0 (100)	
G	G1	46 (66.7)	23 (33.3)	69 (100)	0.028
	G2	13 (40.6)	19 (59.4)	32 (100)	
	G3	44 (65.7)	23 (34.3)	67 (100)	
	(Missing)	0 (0.0)	7 (100.0)	7 (100)	
Bd	Bd0	1 (50.0)	1 (50.0)	2 (100)	0.383
	Bd1	47 (64.4)	26 (35.6)	73 (100)	
	Bd2	14 (48.3)	15 (51.7)	29 (100)	
	Bd3	23 (67.6)	11 (32.4)	34 (100)	
	(Missing)	18 (48.6)	19 (51.4)	37 (100)	
LVI	LVI−	44 (58.7)	31 (41.3)	75 (100)	0.513
	LVI+	59 (64.8)	32 (35.2)	91 (100)	
	(Missing)	0 (0.0)	9 (100.0)	9 (100)	
MSI	MSS	52 (58.4)	37 (41.6)	89 (100)	<0.001
	MSI-L	23 (100.0)	0 (0.0)	23 (100)	
	MSI-H	9 (90.0)	1 (10.0)	10 (100)	
	(Missing)	19 (35.8)	34 (64.2)	53 (100)	

**Table 4:** Variables distributions in relation to LNM.

Variable		Right				Left				Total			
		pN0	pN+	Total	*p*	pN0	pN+	Total	*p*	pN0	pN+	Total	*p*
pT	pTis	1 (1.5)	0 (0.0)	1 (1.0)	<0.001	0 (0.0)	0 (0.0)	0 (0.0)	0.023	1 (0.9)	0 (0.0)	1 (0.6)	<0.001
	pT1	11 (16.7)	0 (0.0)	11 (10.7)		11 (26.8)	2 (6.5)	13 (18.1)		22 (20.6)	2 (2.9)	24 (13.7)	
	pT2	7 (10.6)	1 (2.7)	8 (7.8)		8 (19.5)	2 (6.5)	10 (13.9)		15 (14.0)	3 (4.4)	18 (10.3)	
	pT3	39 (59.1)	15 (40.5)	54 (52.4)		15 (36.6)	16 (51.6)	31 (43.1)		54 (50.5)	31 (45.6)	85 (48.6)	
	pT4	8 (12.1)	21 (56.8)	29 (28.2)		7 (17.1)	11 (35.5)	18 (25.0)		15 (14.0)	32 (47.1)	47 (26.9)	
G	G1	34 (51.5)	12 (32.4)	46 (44.7)	0.009	15 (36.6)	8 (25.8)	23 (31.9)	0.489	49 (45.8)	20 (29.4)	69 (39.4)	0.013
	G2	11 (16.7)	2 (5.4)	13 (12.6)		11 (26.8)	8 (25.8)	19 (26.4)		22 (20.6)	10 (14.7)	32 (18.3)	
	G3	21 (31.8)	23 (62.2)	44 (42.7)		11 (26.8)	12 (38.7)	23 (31.9)		32 (29.9)	35 (51.5)	67 (38.3)	
	(Miss.)	0 (0.0)	0 (0.0)	0 (0.0)		4 (9.8)	3 (9.7)	7 (9.7)		4 (3.7)	3 (4.4)	7 (4.0)	
Bd	Bd0	1 (1.5)	0 (0.0)	1 (1.0)	0.012	1 (2.4)	0 (0.0)	1 (1.4)	0.004	2 (1.9)	0 (0.0)	2 (1.1)	<0.001
	Bd1	35 (53.0)	12 (32.4)	47 (45.6)		20 (48.8)	6 (19.4)	26 (36.1)		55 (51.4)	18 (26.5)	73 (41.7)	
	Bd2	9 (13.6)	5 (13.5)	14 (13.6)		6 (14.6)	9 (29.0)	15 (20.8)		15 (14.0)	14 (20.6)	29 (16.6)	
	Bd3	8 (12.1)	15 (40.5)	23 (22.3)		2 (4.9)	9 (29.0)	11 (15.3)		10 (9.3)	24 (35.3)	34 (19.4)	
	(Miss.)	13 (19.7)	5 (13.5)	18 (17.5)		12 (29.3)	7 (22.6)	19 (26.4)		25 (23.4)	12 (17.6)	37 (21.1)	
LVI	LVI−	41 (62.1)	3 (8.1)	44 (42.7)	<0.001	25 (61.0)	6 (19.4)	31 (43.1)	0.001	66 (61.7)	9 (13.2)	75 (42.9)	<0.001
	LVI+	25 (37.9)	34 (91.9)	59 (57.3)		12 (29.3)	20 (64.5)	32 (44.4)		37 (34.6)	54 (79.4)	91 (52.0)	
	(Miss.)	0 (0.0)	0 (0.0)	0 (0.0)		4 (9.8)	5 (16.1)	9 (12.5)		4 (3.7)	5 (7.4)	9 (5.1)	
MSI	MSS	34 (51.5)	18 (48.6)	52 (50.5)	0.747	21 (51.2)	16 (51.6)	37 (51.4)	1.000	55 (51.4)	34 (50.0)	89 (50.9)	0.451
	MSI-L	16 (24.2)	7 (18.9)	23 (22.3)		0 (0.0)	0 (0.0)	0 (0.0)		16 (15.0)	7 (10.3)	23 (13.1)	
	MSI-H	7 (10.6)	2 (5.4)	9 (8.7)		1 (2.4)	0 (0.0)	1 (1.4)		8 (7.5)	2 (2.9)	10 (5.7)	
	(Miss.)	9 (13.6)	10 (27.0)	19 (18.4)		19 (46.3)	15 (48.4)	34 (47.2)		28 (26.2)	25 (36.8)	53 (30.3)	

**Table 5:** Multivariate analysis.

**Right**					
**Variable**		**pN0**	**pN+**	**OR (Univariate)**	**OR (Multivariate)**
pT	pTis, pT1–3	58 (78.4)	16 (21.6)	-	-
	pT4	8 (27.6)	21 (72.4)	9.52 (3.68–26.82, *p* < 0.001)	8.19 (1.62–63.58, *p* = 0.019)
G	G1–2	45 (76.3)	14 (23.7)	-	-
	G3	21 (47.7)	23 (52.3)	3.52 (1.54–8.35, *p* = 0.003)	3.24 (0.81–14.66, *p* = 0.106)
Bd	Bd1–2	45 (72.6)	17 (27.4)	-	-
	Bd3	8 (34.8)	15 (65.2)	4.96 (1.83–14.41, *p* = 0.002)	2.08 (0.47–9.97, *p* = 0.340)
LVI	LVI−	41 (93.2)	3 (6.8)	-	-
	LVI+	25 (42.4)	34 (57.6)	18.59 (5.90–82.96, *p* < 0.001)	25.96 (3.80–570.24, *p* = 0.006)
MSI	MSS, MSI-L	50 (66.7)	25 (33.3)	-	-
	MSI-H	7 (77.8)	2 (22.2)	0.57 (0.08–2.57, *p* = 0.504)	0.98 (0.06–14.03, *p* = 0.989)
**Left**					
**Variable**		**pN0**	**pN+**	**OR (Univariate)**	**OR (Multivariate)**
pT	pTis, pT1–3	34 (63.0)	20 (37.0)	-	-
	pT4	7 (38.9)	11 (61.1)	2.67 (0.91–8.34, *p* = 0.079)	0.75 (0.06–8.46, *p* = 0.808)
G	G1–2	26 (61.9)	16 (38.1)	-	-
	G3	11 (47.8)	12 (52.2)	1.77 (0.64–5.04, *p* = 0.275)	0.90 (0.12–6.05, *p* = 0.910)
Bd	Bd1–2	27 (64.3)	15 (35.7)	-	-
	Bd3	2 (18.2)	9 (81.8)	8.10 (1.80–57.90, *p* = 0.013)	5.37 (0.69–66.73, *p* = 0.132)
LVI	LVI−	25 (80.6)	6 (19.4)	-	-
	LVI+	12 (37.5)	20 (62.5)	6.94 (2.32–23.36, *p* = 0.001)	11.53 (1.45–247.78, *p* = 0.041)
MSI	MSS, MSI-L	21 (56.8)	16 (43.2)	-	-
	MSI-H	1 (100.0)	0 (0.0)	0.00 (Na–>1000, *p* = 0.995)	0.00 (Na–>1000, *p* = 0.995)
**Right + Left**					
**Variable**		**pN0**	**pN+**	**OR (Univariate)**	**OR (Multivariate)**
pT	pTis, pT1–3	92 (71.9)	36 (28.1)	-	-
	pT4	15 (31.9)	32 (68.1)	5.45 (2.69–11.51, *p* < 0.001)	3.46 (0.98–14.23, *p* = 0.063)
G	G1–2	71 (70.3)	30 (29.7)	-	-
	G3	32 (47.8)	35 (52.2)	2.59 (1.37–4.96, *p* = 0.004)	1.94 (0.66–5.86, *p* = 0.229)
Bd	Bd1–2	72 (69.2)	32 (30.8)	-	-
	Bd3	10 (29.4)	24 (70.6)	5.40 (2.37–13.08, *p* < 0.001)	2.45 (0.78–8.10, *p* = 0.130)
LVI	LVI−	66 (88.0)	9 (12.0)	-	-
	LVI+	37 (40.7)	54 (59.3)	10.70 (4.95–25.47, *p* < 0.001)	20.64 (4.99–147.28, *p* < 0.001)
MSI	MSS, MSI-L	71 (63.4)	41 (36.6)	-	-
	MSI-H	8 (80.0)	2 (20.0)	0.43 (0.06–1.83, *p* = 0.304)	0.80 (0.07–7.32, *p* = 0.846)

## Data Availability

The data can be shared up on request.
